# Effectiveness of palbociclib with aromatase inhibitors for the treatment of advanced breast cancer in an exposure retrospective cohort study: implications for clinical practice

**DOI:** 10.1186/s13058-023-01678-5

**Published:** 2023-06-29

**Authors:** Filipa Alves da Costa, Fábio Cardoso Borges, Adriana Ramos, Alexandra Mayer, Claudia Brito, Catarina Ramos, Catarina Bernardo, Mariane Cossito, Cláudia Furtado, Arlindo R. Ferreira, Diogo Martins-Branco, Ana da Costa Miranda, António Lourenço

**Affiliations:** 1 Registo Oncológico Nacional (RON), Instituto Português de Oncologia de Lisboa Francisco Gentil, EPE, Lisbon, Portugal; 2 Research Institute for Medicines (iMED), Faculty of Pharmacy, University of Lisbon, Lisbon, Portugal; 3 Direção de Avaliação de Tecnologias de Saúde, Autoridade Nacional do Medicamento e Produtos de Saúde, I.P. (INFARMED I.P.), Lisbon, Portugal; 4 Unidade de Mama, Centro Clínico Champalimaud, Fundação Champalimaud, Lisbon, Portugal; 5 Católica Medical School, Universidade Católica Portuguesa, Lisbon, Portugal; 6 Serviço de Oncologia Médica, Instituto Português de Oncologia de Lisboa Francisco Gentil, EPE, Lisbon, Portugal; 7 Academic Trials Promoting Team, Institute Jules Bordet, Rue Meylemeersch 90, 1070 Brussels, Belgium; 8 NOVA Medical School, Universidade Nova de Lisboa, Lisbon, Portugal

**Keywords:** Palbociclib, Effectiveness, Safety, Cancer registry

## Abstract

**Background:**

New drugs for locally advanced or metastatic breast cancer have led to clinical benefits, aside with increasing costs to healthcare systems. The current financing model for health technology assessment (HTA) privileges real-world data. As part of the ongoing HTA, this study aimed to evaluate the effectiveness of palbociclib with aromatase inhibitors (AI) and compare it with the efficacy reported in PALOMA-2.

**Methods:**

A population-based retrospective exposure cohort study was conducted including all patients initiating treatment in Portugal with palbociclib under early access use and registered in the National Oncology Registry. The primary outcome was progression free survival (PFS). Secondary outcomes considered included time to palbociclib failure (TPF), overall survival (OS), time to next treatment (TTNT), and proportion of patients discontinuing treatment due to  adverse events (AEs). The Kaplan–Meier method was used and median, 1- and 2-year survival rates were computed, with two-sided 95% confidence intervals (95%CI). STrengthening the Reporting of OBservational studies in Epidemiology (STROBE) guidelines for reporting observational studies were used.

**Results:**

There were 131 patients included. Median follow-up was 28.3 months (IQR: 22.7–35.2) and median duration of treatment was 17.5 months (IQR: 7.8–29.1). Median PFS was 19.5 months (95%CI 14.2–24.2), corresponding to a 1-year PFS rate of 67.9% (95%CI 59.2–75.2) and a 2-year PFS rate of 42.0% (95%CI 33.5–50.3). Sensitivity analysis showed median PFS would increase slightly when excluding those not initiating treatment with the recommended dose, raising to 19.8 months (95%CI 14.4–28.9). By considering only patients meeting PALOMA-2 criteria, we could observe a major difference in treatment outcomes, with a mean PFS of 28.8 months (95%CI 19.4–36.0). TPF was 19.8 months (95%CI 14.2–24.9). Median OS was not reached. Median TTNT was 22.5 months (95%CI 18.0–29.8). A total of 14 patients discontinued palbociclib because of AEs (10.7%).

**Conclusions:**

Data suggest palbociclib with AI to have an effectiveness of 28.8 months, when used in patients with overlapping characteristics to those used in PALOMA-2. However, when used outside of these eligibility criteria, namely in patients with less favorable prognosis (e.g., presence of visceral disease), the benefits are inferior, even though still favorable.

**Supplementary Information:**

The online version contains supplementary material available at 10.1186/s13058-023-01678-5.

## Background

The emergence of new drugs has led to breakthroughs in the management of breast cancer, although with increasing costs to healthcare systems worldwide [1]. New financing models have been developed, some of which incorporating real-world drug performance (especially drug effectiveness) based on post-marketing health technology assessment (HTA) [2, 3]. In the Portuguese National Health Service (that follows a typical Beveridge model), the financing model anticipates that drugs targeting diseases without alternative treatments can be provided for 210 days under an exceptional authorizations’ schema [4] before final reimbursement decision (early access program). During this period, data may be recorded for evaluation of outcomes that will inform future drug re-evaluations, thus opening the opportunity for effectiveness studies [5].

Most breast cancer cases are currently diagnosed at early stages, but metastatic (de novo or after systemic disease recurrence) breast cancer remains an incurable disease with a 5-year survival rate of 33.8% [6]. It is argued that recent improvements in overall survival (OS) were mostly due to advances in the treatment of patients with human epidermal growth factor receptor 2 (HER2)-positive advanced breast cancer (ABC) [7, 8]. Cyclin-dependent kinase 4/6 (CDK4/6) inhibitors are a relatively new class of drugs that redefined the standard of care for patients with endocrine receptor (ER)-positive, HER2-negative ABC [9]. Palbociclib, the first of this new class of agents, received marketing authorization in the European Union in 2016 for the treatment of ER-positive, HER2-negative, locally advanced or metastatic breast cancer. Palbociclib is currently available in combination with an aromatase inhibitor (AI) or in combination with fulvestrant in patients who have received prior endocrine therapy. The approval in combination with an AI was based on PALOMA-2, a double-blind, phase 3 randomized controlled trial that included 666 post-menopausal women with ER-positive/HER2-negative ABC with no prior systemic therapy for advanced disease to receive palbociclib plus letrozole or placebo plus letrozole [10]. This trial demonstrated benefit for the combination, with a hazard ratio (HR) for disease progression or death (primary outcome) of 0.6 (95% confidence interval [CI] 0.5–0.7; *p* < 0.001). A recent study update, with a 90-month follow-up, confirmed favorable results for PFS but shorter OS, despite not significant (44.6 compared to 51.6 in the placebo arm; HR: 0.9 [95% CI 0.7–1.1]) [11]. All-causality treatment-emergent adverse events (AEs) led to the discontinuation of palbociclib-letrozole in 9.7% of patients in the trial [10]. A slightly higher proportion, 12.2%, was reported in a subsequent sub analysis conducted in 444 women with extended follow-up of 37.6 months [12]. No differences in quality of life were recorded between study arms. [12]. Despite these results, there is a need to evaluate the extent to which these data are transferrable to clinical practice outside the controlled environment of clinical trials [13, 14]. Of note, while PALOMA-2 only included post-menopausal women, the European Medicines Agency (EMA) extends the indication to pre-menopausal women receiving concomitantly a luteinizing hormone-releasing hormone agonist, further increasing the need for real-world evaluations.

Real-world studies evaluating the effectiveness of palbociclib combined with AIs have been recently conducted. In some of these studies, lower effectiveness as assessed by PFS has been reported [15], whereas others found higher effectiveness [16, 17]. Other studies, considering the limited time of follow-up, were unable to estimate median PFS [18, 19].

Considering the current post-marketing HTA model in Portugal, the development of a study with real-world data was necessary to provide evidence that could inform final reimbursement decision.

## Methods

### Aim

The aim of this study was to evaluate the effectiveness of palbociclib in association with an AI in an exposure cohort of patients with ER-positive/HER2-negative breast cancer and to explore determinants of differential effectiveness. We hypothesize that, despite the typical efficacy-effectiveness gap, the real-world effectiveness of palbociclib in combination with an AI will be similar to the efficacy reported in PALOMA-2.

### Study design

A population-based, retrospective, non-comparative, exposure cohort study was conducted including all patients initiating treatment in Portugal with palbociclib between 31/05/2017 and 31/03/2019 that were registered in the National Oncology Registry (Registo Oncológico Nacional; RON). The study period was defined considering the interval in which an early access program for palbociclib was active. We were also able to retrieve information from patients treated in private institutions (excluded from such programs). The follow-up extended from day of treatment initiation until date of last known contact or cut-off date, established on 28/02/2021. STrengthening the Reporting of OBservational studies in Epidemiology (STROBE) guidelines for reporting observational studies were used [20].

### Eligibility criteria

We included adult patients (aged 18 or older), with an established ABC diagnosis (locally advanced unresectable or metastatic breast cancer), that were enrolled in the early access program for palbociclib, or in the case of private institutions, initiating the same treatment between 31/05/2017 and 31/03/2019. The patients must have received at least one administration of palbociclib in association with an AI (anastrozole, letrozole or exemestane). No exclusion criteria were considered.

### Data sources

RON, a nationwide population-based cancer registry, was used as the primary data source. Cancer cases are identified through institution-based databases (mainly from pathology departments) and numerous variables are semi-automatically transferred into the database (e.g., date of birth, sex, place of residence, topography, morphology, vital status and date of death). Other relevant data (e.g., disease stage, prognostic features, and treatments) are registered manually at each health institution, by trained and experienced personnel. For this study, cases of interest were identified using RON, but also complemented with the National Authority of Medicines and Health Products (INFARMED, I.P.) database (the institution approving eligibility for early access programs) to ensure the exhaustiveness of cases. When required, local pharmacies and oncology departments were also contacted to ensure treatment and clinical variables’ exhaustiveness. Information of interest included: (a) demographic and clinical characteristics [sex, age, stage at diagnosis, morphology, stage at treatment initiation, metastasis location at treatment initiation, Eastern Cooperative Oncology Group Performance Status (ECOG PS) at treatment initiation, ER status, HER2 status, previous therapies for advanced disease]; (b) palbociclib plus AI exposure (treatment initiation date, concomitant therapies, dose, dose reduction, date and reason for treatment discontinuation, AEs leading to treatment discontinuation); (c) outcomes and post-treatment characterization (disease progression and date, subsequent treatments, vital status and date of last known contact/death). RON coordinated contacts with local sites and INFARMED to ensure timely update and exhaustiveness of case reporting.

### Outcome measures

To assess treatment effectiveness, the primary outcome was progression-free survival (PFS). PFS was defined as time from treatment initiation to date of disease progression or death from any cause. Disease progression was defined as radiological progression, clinical progression, or initiation of a new treatment line for ABC (in this hierarchical order of importance). Secondary outcomes were time to palbociclib failure (TPF), overall survival (OS), time to next treatment (TTNT), and proportion of patients discontinuing treatment due to AEs. AEs leading to treatment discontinuation were coded according to The Medical Dictionary for Regulatory Activities [21]. TPF was defined as the time from palbociclib initiation to date of treatment discontinuation due to any cause. OS was defined as the time from treatment initiation to date of death from any cause. TTNT was defined as the time from initiation of palbociclib until the initiation of a new treatment (irrespective of the reason). For all time-to-event outcomes (PFS, TPF, OS and TTNT), patients who were alive without the event of interest were censored at the last known contact/data cut-off. Treatments considered included chemotherapy, endocrine therapy or targeted therapy. As menopausal status is not collected systematically by RON, the following cut-offs were considered taking previous studies in the Portuguese population: pre-menopausal women as those ≤ 50, peri menopausal as those between 50 and 55 and post-menopausal as those aged over 55 years [22]. Metastatic locations were classified in three different groups: visceral (those located in lung/pleura, liver, peritoneum or brain), bone-only (those exclusively located in the bone) and non-visceral metastases (those not fitting into any of the former categories).

### Sources of bias

This study focused on the use of palbociclib under an early access program, previous to reimbursement decision, which may potentially lead to channeling bias. Despite the best efforts to obtain adverse event data, the use of a cancer registry as the main data source limits the ability to fully characterize all AEs. In addition, the retrospective nature of the study may lead to misclassification bias for various outcomes, including PFS and AEs. In a real-world context, assessment of disease progression is not done at the same intervals as in interventional studies, thus potentially leading to overestimation of PFS. The same applies to the reporting of AEs, which is less rigorous in a real-world context, resulting on a potential underreporting, reason why we focused on AEs leading to treatment discontinuation.

### Statistical analysis

Data registered in RON was exported in a pseudo-anonymized format for analysis. Data were validated considering missing data and cross-variable validation prior to analysis. Patient characteristics were analyzed using descriptive statistics with measures of central tendency (median) and dispersion (range and quartiles) for continuous variables, and absolute and relative frequencies for categorical variables. Time to event outcomes were evaluated using the Kaplan–Meier method. For each time to event outcome, estimates of the median, one and two-year survival rates were computed, with corresponding two-sided 95% confidence intervals (95%CI). Sample size and power calculations were not performed, considering the population-based nature of the study. Subgroup analysis was conducted to evaluate if outcomes behaved differently in subsets of patients of clinical interest and with n ≥ 30 (de novo metastatic versus recurrent ABC, and location of distant metastasis at treatment initiation). Additional sensitivity analyses were conducted taking in consideration specific PALOMA-2 trial eligibility criteria, namely full initial dose, patients not switching AI during treatment, patients without previous exposure to any systemic treatment to advanced disease, patients without disease recurrence while receiving neoadjuvant or adjuvant therapy with AI or within 12 months after completing this therapy, post-menopausal status, and patients with radiologically assessed disease progression [23]. For all sensitivity analyses, unknown or not evaluated subgroups were disregarded. Multivariate proportional hazard regression was conducted for computing hazard ratios, and presented using forest plots, considering covariates statistically significant in univariate analysis or whose clinical relevance for prognostic value is undisputed. Covariates presenting ≥ 25% unknown responses were excluded. AEs leading to discontinuation were analyzed descriptively. To note that the same patient may have experienced more than one AE leading to treatment discontinuation. Statistical analyses were performed using Stata software, version 13.0 [24].

## Results

During the study period, we identified 131 patients receiving palbociclib in association with an AI. All individuals included in the cohort were women and had a median age at treatment initiation of 58 years [interquartile range (IQR): 47–67]. Median time elapsed from diagnosis to initiation of treatment was 3.9 months (IQR: 0.6–9.5). Upon initiation of treatment, nearly all individuals had metastatic disease (98.5%), among which 55.8% had visceral metastases. Among patients with metastatic disease, 51 patients had de novo metastasis (39.5%) and 78 had recurrent metastasis (60.5%). Also, the vast majority had ECOG PS of 0 or 1 (94.7%), most were post-menopausal (67.2%) and all had ER-positive and HER2-negative tumors. Socio-demographic and clinical characteristics of the exposure cohort are presented in comparison with those reported for PALOMA-2 trial participants (Table 1).

**Table 1:** Patients’ socio-demographic and clinical characteristics at baseline

Characteristics		RON (*n* = 131)	PALOMA-2 (*n* = 444)
Simplified staging (TNM) at diagnosis, *n* (%)	I–III	80 (61.1)	260 (58.5)
	IV	51 (38.9)	138 (31.1)
	Unknown/missing	0 (0.0)	46 (10.4)
Histologic subtype, *n* (%)	No special type invasive carcinoma	109 (83.2)	NA
	Invasive lobular carcinoma tumor	13 (9.9)	NA
	Other	9 (6.9)	NA
Age at initiation of treatment	Median (IQR 25–75)	58 (47–67)	62 (NA)
	Min–max	27–80	30–89
Disease extension and location of metastasis upon initiation of treatment, *n* (%)	M0	2 (1.5)	0 (0.0)
	M1	129	444 (100.0)
	Visceral	(98.5)	214 (48.2)
	Non visceral	72 (55.8)	230 (51.8)
	Bone-only	19 (14.7)	103 (23.2)^$^
		38 (29.5)	
Performance status at treatment initiation, *n* (%)	0–1	108 (94.7)	435 (98.0)
Unknown: *n* = 17	≥ 2	6 (5.3)	9 (2.0)
Menopausal status	Pre or peri menopausal	43 (32.8)	0 (0.0)
	Post-menopausal	88 (67.2)	444 (100.0)
Hormonal receptors status, *n* (%)*	Positive estrogen and/or progestogen receptors	129 (100.0)	444 (100.0)
*Not evaluated/unknown n* = *2*	Negative estrogen and progestogen receptors	0 (0.0)	0 (0.0)
HER2 Status, *n* (%)#	Positive	0 (0.0)	0 (0.0)
*Not evaluated/unknown n* = *6*	Negative	125 (100.0)	444 (100.0)

*HER2*, Human epidermal growth factor receptor 2, *IQR*, Interquartile range, *NA*, not available

*Information obtained for 76 patients upon diagnosis and for 55 patients upon treatment initiation

^**#**^Information obtained for 72 patients upon diagnosis and for 59 patients upon treatment initiation

^$^In PALOMA-2, the category “bone-only” is a subcategory of “non-visceral metastases”, whereas in the current study “bone-only” was considered an independent category and therefore these data are not directly comparable

The median follow-up of the patients included in the study was 28.3 months (IQR: 22.7–35.2), with a median duration of treatment of 17.5 months (IQR: 7.8–29.1), which corresponds to a median of 16 cycles of treatment. There were 11 patients lost to follow-up (8.4%). The median time elapsed since diagnosis of breast cancer and initiation of palbociclib was 3.9 years (IQR: 0.6–9.5). Prior to palbociclib + AI initiation, around a quarter of patients (*n* = 35 patients; 26.7%) had not received any systemic therapy for either locoregional or advanced disease and 96 patients (73.3%) had received at least one line of such therapy. The description of the number of lines of therapy in the context of locoregional or advanced disease is included in Table 2 and a detailed description of previous lines of therapy is available as Additional file 1: Table S1. At the time of study, most patients were taking palbociclib in association with letrozole (80.9%). Nearly all patients initiated palbociclib (94.4%) using the dose of 125 mg, even though 52.6% reduced the dose subsequently. At the cut-off date, 40 patients were still on treatment. The most common reason for treatment discontinuation was disease progression (73.6%) (Table 2).

**Table 2:** Treatment characteristics

Follow-up time, median (months)	28.3
Completeness of follow-up (%)	91.6
*Time elapsed since diagnosis and initiation of palbociclib (years)*	
Median (IQR)	3.9 (0.6–9.5)
Min–max	0.0–23.9
Previous lines of treatment for locoregional or advanced disease*	
0 lines, *n* (%)	35 (26.7)
1 line, *n* (%)	58 (44.3)
2 lines, *n* (%)	18 (13.7)
3 lines, *n* (%)	13 (9.9)
4 lines, *n* (%)	5 (3.8)
≥ 5 lines, *n* (%)	2 (1.6)
*Concurrent AI*	
Anastrazole, *n* (%)	15 (11.5)
Exemestane, *n* (%)	10 (7.6)
Letrozole, n (%)	106 (80.9)
*Other treatments received concurrently to palbociclib + AI, n (%)*	
LHRH agonist	31 (23.7)
Bisphosphonates or monoclonal antibody anti- RANK/RANKL	76 (58.0)
Radiotherapy	26 (19.9)
Surgery	9 (6.9)
Duration of palbociclib treatment, months, median (IQR)	17.53 (7.8– 29.1)
Number of palbociclib cycles, median (IQR)	16 (7–29)
*Initial dose (mg) of palbociclib, n (%) [Unknown, n = 6]*	
125 mg	118 (94.4)
100 mg	7 (5.6)
75 mg	0 (0.0)
*Reduced initial dose of palbociclib, n (%) [Unknown, n = 55]*	
Yes	40 (52.6)
No	36 (47.4)
*Treatment status at cut-off date, n (%)*	
Patients with palbociclib treatment on-going at cut-off date	40 (30.5)
Discontinued treatment	91 (69.5)
*Reasons for treatment discontinuation*	
Disease progression	67 (73.6)
Death	1 (1.1)
Adverse event	14 (15.4)
Patient refusal	2 (2.2)
Other cause	4 (4.4)
Unknown reason	3 (3.3)
*Continuation of treatment (n = 91)*	
No systemic treatment after palbociclib	12 (13.2)
One or more subsequent systemic treatments	79 (86.8)

*AI*, aromatase inhibitor; *IQR*, Interquartile range; *LHRH*, Luteinizing hormone releasing hormone; *RANK/RANKL*, Receptor activator of NF-κB ligand;

*Patients may not have received any previous treatment and those that have received, may have undergone various treatments; therefore, we present the lines of treatment, irrespective of their nature; 58 patients received exclusively adjuvant endocrine therapy for locoregional disease at diagnosis, 9 received exclusively endocrine therapy for advanced disease and another 9 received exclusively chemotherapy for advanced disease. Further details about previous treatments are available as Additional file 1: Table S1

**Fig. 1:**
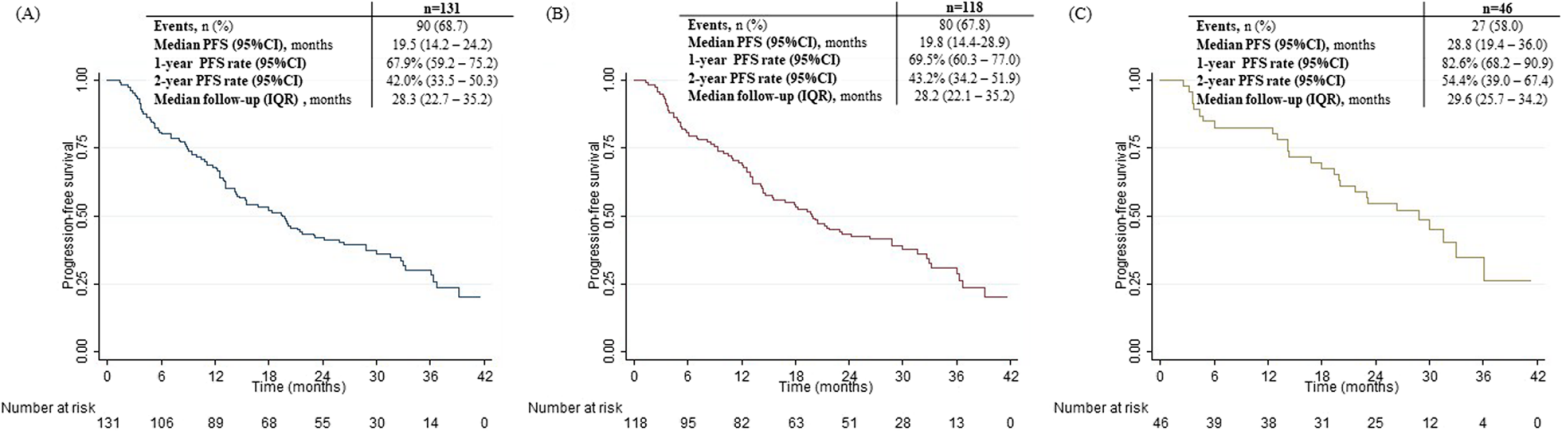
Sensitivity analysis for PFS; **A**: all patients; **B**: excluding patients not initiating treatment with the recommended dose; **C**: excluding patients not meeting all the selected PALOMA-2 criteria

Following palbociclib + AI permanent discontinuation, most patients (*n* = 79; 86.8%) received subsequent systemic anticancer therapies. Among these, 34 did so due to disease progression. Chemotherapy was the most common first subsequent treatment, followed by endocrine therapy and targeted therapies. An important number of patients received a second subsequent therapy (*n* = 45), 28.9% of which following disease progression (*n* = 13), and some received a third or more subsequent therapies (*n* = 28), 14.3% of which following disease progression (*n* = 4) (data not shown). Median PFS was 19.5 months (95%CI 14.2–24.2), corresponding to a 1-year PFS rate of 67.9% (95%CI 59.2–75.2) and a 2-year PFS rate of 42.0% (95%CI 33.5–50.3) (Fig. 1).

Sensitivity analysis showed median PFS would increase slightly when excluding those not initiating treatment with the recommended dose, raising to 19.8 months (95%CI 14.4–28.9), corresponding to a 1-year PFS rate of 69.5% (95%CI 60.3–77.0) and a 2-year PFS rate of 43.2% (95%CI 34.2–51.9) (Fig. 1).

The sensitivity analyses according to selected inclusion criteria of PALOMA-2 identified two characteristics that led to a considerable difference in PFS. By excluding patients with clinically evaluated progression, we could observe a gain of nearly 2 months, and by excluding patients with previous exposure to at least one systemic treatment line to advanced disease and those with disease recurrence while receiving neoadjuvant or adjuvant therapy with AI or within 12 months after completing this therapy, we could observe a gain of around 3.5 months. By considering only patients meeting all the selected PALOMA-2 criteria, we could observe a major difference in treatment outcomes, with a mean PFS of 28.8 (19.4–36.0), 1-year PFS rate of 82.6% and 2-year PFS-rate of 54.4% (Fig. 1 and Additional file 1: Table S2).

**Fig. 2:**
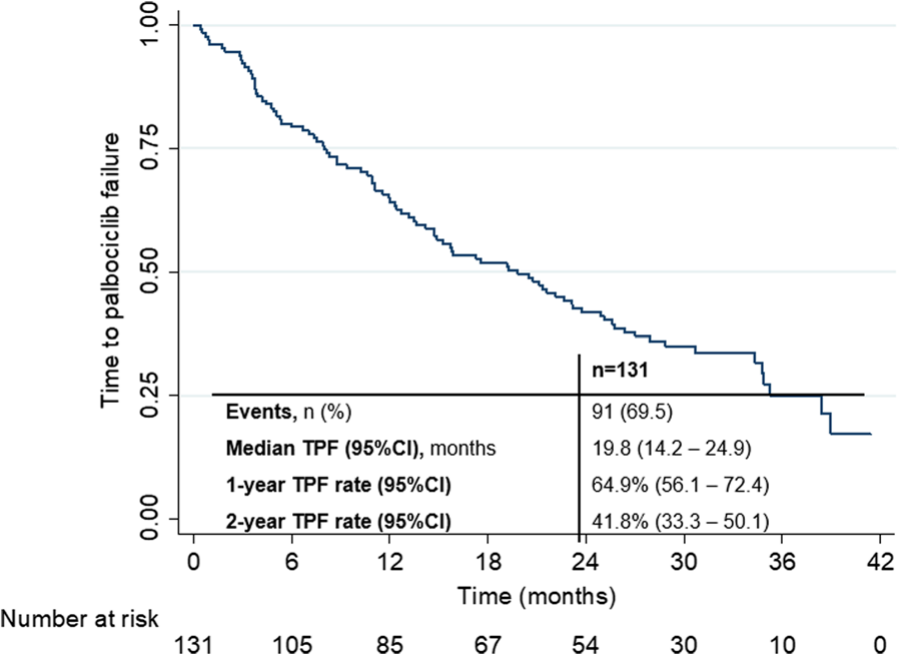
Secondary endpoint—time to palbociclib failure (TPF)

By the cut-off date, a total of 91 patients had discontinued palbociclib from any cause. Median TPF was 19.8 months (95%CI 14.2–24.9), corresponding to a 1-year TPF rate of 64.9% (95%CI 56.1–72.4) and a 2-year TPF rate of 41.8% (95%CI 33.3–50.1). (Fig. 2).

**Fig. 3:**
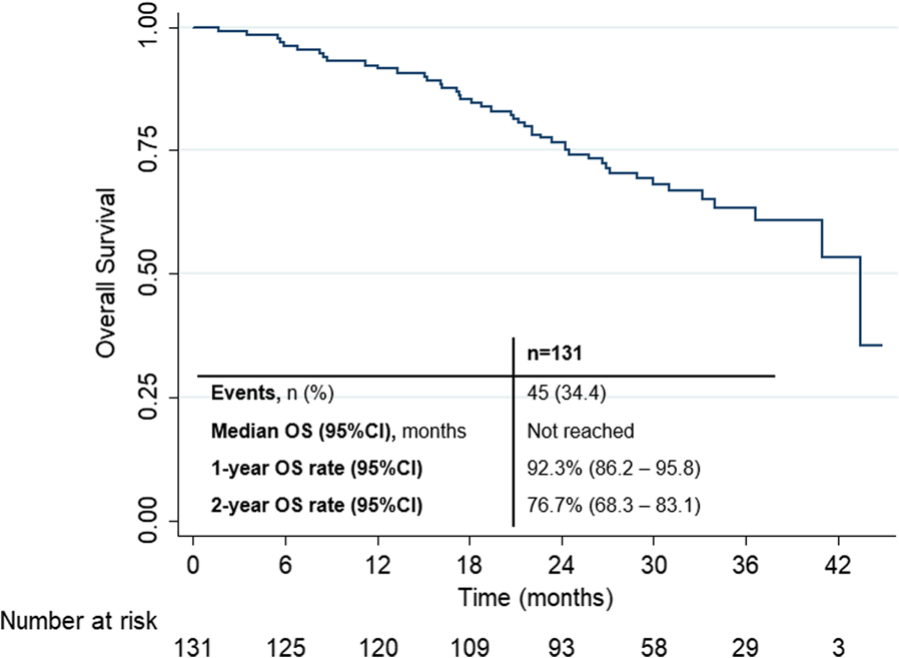
Secondary endpoint—overall survival (OS)

A total of 45 death events had occurred (34.4%). Median OS was not reached. The 1-year and 2-year OS rate were, respectively, 92.3% (95%CI 86.2–95.8) and 76.7% (95%CI 68.3–83.1) (Fig. 3).

**Fig. 4:**
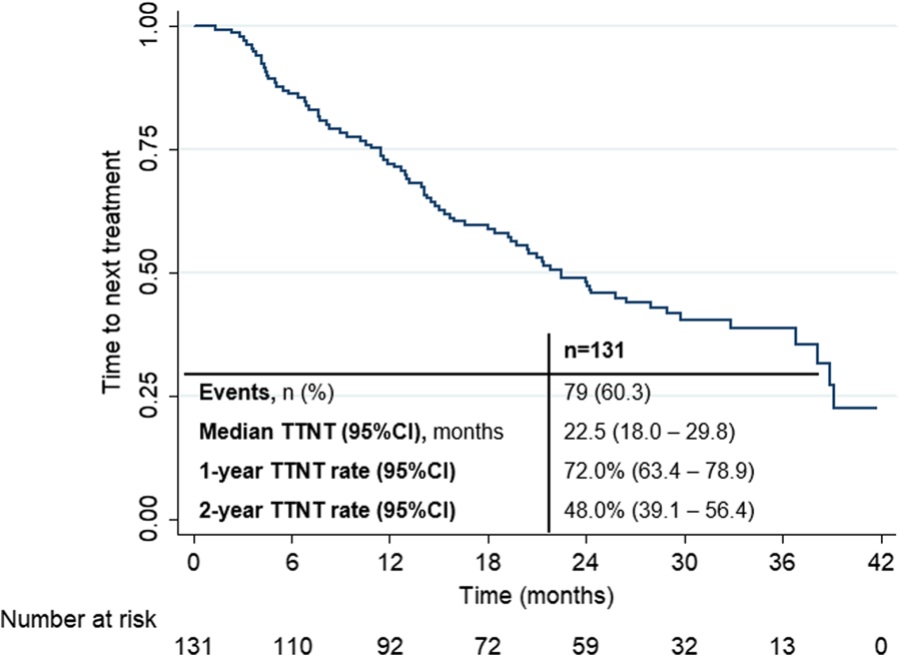
Secondary endpoint—time to next treatment (TTNT)

Seventy-nine patients (60.3%) were switched to another treatment. Median TTNT was 22.5 months (95%CI 18.0–29.8), corresponding to a 1-year TTNT rate of 72.0% (95%CI 63.4–78.9) and a 2-year TTNT rate of 48.0% (95%CI 39.1–56.4) (Fig. 4).

A total of 14 patients discontinued palbociclib because of AEs (10.7%). The most common AEs leading to treatment discontinuation were neutropenia [*n* = 8 in total; either as isolated AE, including neutropenia (*n* = 3) and febrile neutropenia (*n* = 1), or in combination with other AEs, including hepatotoxicity (*n* = 2), leucopenia and thrombocytopenia (*n* = 1) and hepatoxicity and anemia (*n* = 1)]. All other AEs were only observed in one patient (data not shown).

Subgroup analyses of PFS according to stratification factors and other baseline characteristics confirmed the findings of the primary analysis. The only factor identified as consistently leading to poorer results was the presence of visceral metastases (15.5 months vs 20.4 months for bone only), a well-established prognostic factor (see Additional file 1: Table S3).

**Fig. 5:**
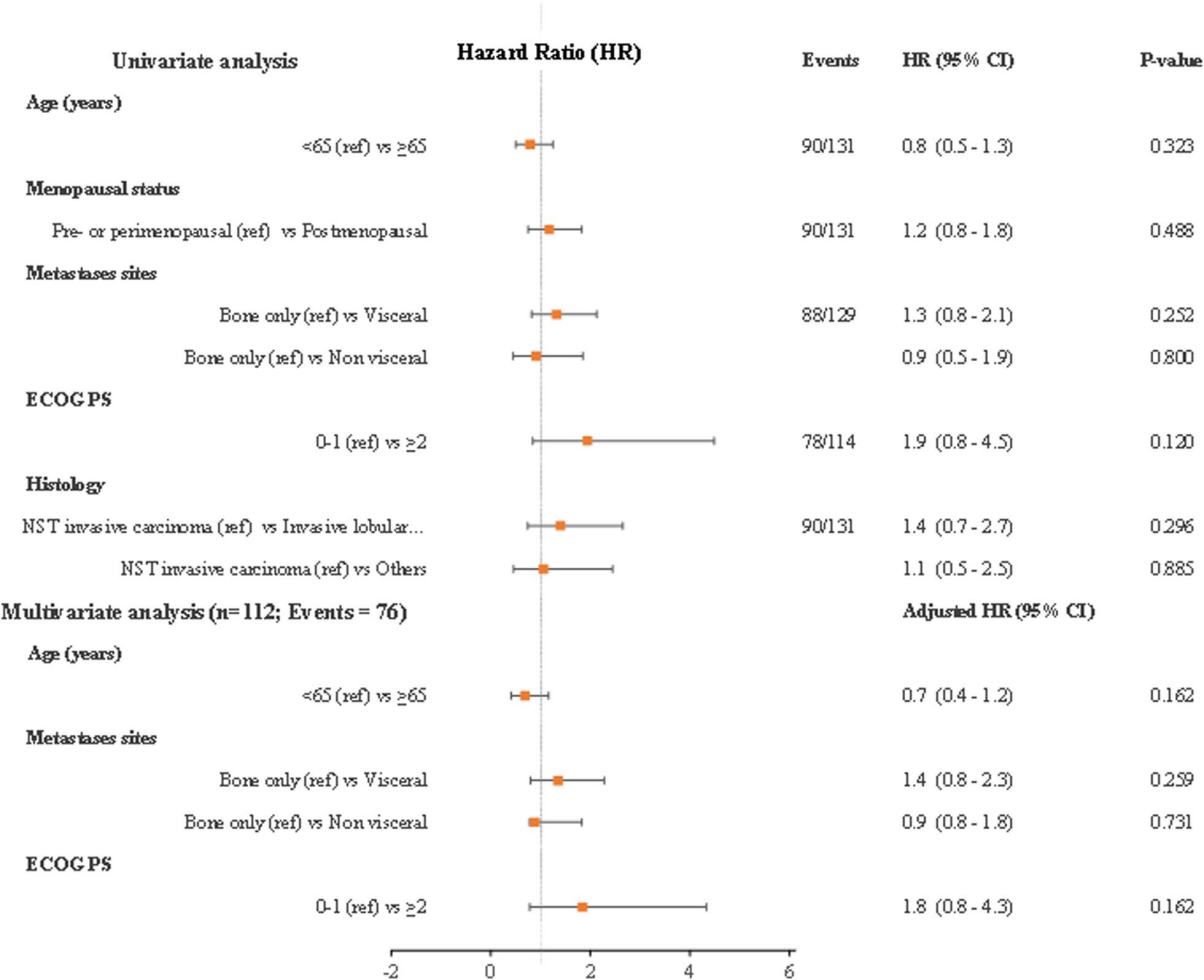
Univariate and multivariate analysis for the primary endpoint, PFS

Covariates of interest kept in the final multivariate model were age group, menopausal status, ECOG PS, histological type and metastatic site upon palbociclib initiation. Multivariate analysis did not reveal any independent factors for an increased risk of disease progression as the three factors included in the model (age, metastatic sites and ECOG PS) did not reach statistical significance (Fig. 5).

## Discussion

This study provides important additional information to the body of evidence available, as it is the first population-based study exploring the effectiveness of palbociclib in association with an AI for the treatment of ABC. In comparison with PALOMA-2 trial, this real-world study enabled the assessment of this therapy’s effect in a population with slightly different characteristics, namely in younger females (58 vs 62 years) but with worse prognostics as suggested by a higher proportion of ECOG PS ≥ 2 (5.3% vs 2.0%), stage IV (38.9% vs 34.7%), with visceral involvement (55.8% vs 49.4%) and de novo metastatic upon treatment initiation (39.5% vs 37.6%) [10].

Median PFS observed in our study was 19.5 months, around five months less than reported in the first presentation of PALOMA-2, 24.8 months [10], and eight months less than the most recent update, 27.6 months [12]. Our findings are similar to previous real-world studies [25] and more favorable than reported elsewhere [15, 26]. Other authors have explored the differential effect when used as first, second or third line (or over) of treatment on PFS [15, 26]. Even though our follow-up period was shorter than PALOMA-2, to our knowledge, no real-world study has been published to date with a longer duration of follow-up [15, 17, 25, 26]. Other studies have additional pitfalls such as relying on retrospective chart reviews [16, 18, 19] (Table 3).

**Table 3:** Real-world effectiveness of palbociclib–summary of evidence available

References	Design	Follow-up	Setting	*n*	Results
Torres et al. [27]	Retrospective multicentric cohort study	Median: 19.4 months	First line (palbo + AI)	878	Median PFS: 21.9 months (95%CI 20.1–28.2)
Varella et al. [15]	Retrospective single-center cohort study	Median: 10.2 months	First line (palbo + AI)	57	Median PFS: 15.1 months (95%CI 12.3-NR)
Taylor-Stokes et al. [18]	Retrospective multicentric cohort study	Mean: 9.9 months (SD 5.2)	First line (palbo + AI)	360	PFS at 12 months: 84.1% OS at 12 months: 95.1%
Wilkie et al. [16]	Retrospective single-center cohort study	Not available	First line (palbo + AI)	70	Median PFS: 26.4 months (95%CI 19.7–33.2)
Waller et al. [19]	Retrospective multicentric cohort study	Mean: 10 months (SD 4)	First line (palbo + AI)	105	PFS at 6 months: 94.0% OS at 6 months: 98.0%
DeMichelle et al.[25]	Retrospective multicentric cohort study	Mean: 24.2 months (IQR, 14.2–34.9)	First line (palbo + AI)	1430	Median PFS: 20.0 months OS not reached
Palumbo et al. [26]	Prospective multicentric cohort study	Mean: 24 months (range 6–32)	First line (palbo + AI)	182	Median PFS: 14.0 months Median OS: 25.0 months

Palbo + AI, Palbociclib in association with an aromatase inhibitor; SD, Standard deviation; IQR, interquartile range; NR, not reached

In addition to the study design and the limitations acknowledged by authors, often there are important differences in patients’ characteristics that may justify results. For instance, a retrospective chart review reporting a higher PFS than our study (21.9 months) seems to be justified by a considerably lower proportion of patients with stage IV (22.2% vs 38.9%) and with visceral involvement (14.8% vs 55.8%) [27]. Also, previous exposure to other AI may impact on findings. This has led us to conduct a sensitivity analysis through which we could observe the effect of the combination when used in similar conditions as those established by EMA, i.e., by excluding patients to whom a dose different from the recommended 125 mg dose was administered. This restriction resulted in a change of PFS from 19.5 to 19.8 months, which may be considered a neglectable effect. Therefore, we have expanded our sensitivity analyses to observe the effect of the combination when used in a situation closer to the PALOMA-2 eligibility criteria. When restricting to patients with a more endocrine sensitive disease (patients with no previous therapy for advanced disease nor disease recurrence while on adjuvant AI or within 12 months after completing this therapy) the median PFS increased to 23.0 months. Likewise, when restricting the evaluation to patients with radiologically assessed disease progression, the median PFS changed to 21.2 months (95%CI 15.4–30.0). However, the exclusion of clinical progression may lead to immortal time bias considering that this excludes those patients with rapidly progressive disease for whom radiological evaluation might not be available before treatment switch.

Above all, when considering all criteria simultaneously, median PFS observed in our study was 28.8 months (median follow-up of 29.6 months), even superior to the estimates reported in the clinical trial (median PFS = 24.8 months; median follow-up of 23 months) and its updated follow-up (median PFS = 27.6 months, median follow-up of 38 months) [10, 12]. These findings reinforce the good performance of palbociclib with an AI for the treatment of ABC, particularly when the treatment is administered under the labelled indications. However, it also underlines that outcomes vary according to patients and disease prognostic characteristics that clinicians should take in consideration when discussing expected results with patients. Nevertheless, there may exist exceptions which need to be considered, such as patients with poor performance status or rapidly progressing disease with visceral crisis, who may not benefit from this treatment. When making such considerations, it is relevant to distinguish features that directly result from transposing patients’ criteria into real life, which are relatively evident, such as hormone-sensitive first-line setting, and those that result from health-care systems’ limitations, such as the use radiological imaging for judging disease progression. Overall, it must be highlighted that the main findings should be put into context of real-world clinical practice. However, the theoretical conditions of the trial are not really that important for ex-post economic evaluation, which aims to capture the costs generated to the system using treatments in a realistic environment. Nonetheless, subgroup analyses identified poorer results in patients with visceral metastases, a well-established prognostic factor that could be considered in differential economic models in the future. However, we do not have effectiveness data for letrozole alone to complete a comparative effectiveness analysis, which would lead to a more contextual read of these results and allow to dissect the identified effectiveness-efficacy gap more clearly.

Median TTNT in our study was 22.5 months, an endpoint not explored in PALOMA-2 neither in previous real-world studies. The overlapping value with PFS is reassuring of the consistency of these findings and supports the label indication of the use of palbociclib until disease progression. A lower median TPF was observed, 19.8 months, roughly 6 months less than reported by Wilkie et al. [16], which may reflect treatment discontinuation due to toxicity.

During the study period, it was not possible to reach a robust estimate of OS given the immaturity of data and therefore additional follow-up is needed. In terms of safety, the proportion of patients discontinuing treatment due to AEs was aligned with PALOMA-2 (10.7% vs 12.2%) [10], even though higher than reported elsewhere [15]. The most common AE reported in PALOMA-2 and in subsequent real-world studies was neutropenia, described to occur in around 60% of cases [10, 15, 16]. In our study, however, even though we found a lower proportion of neutropenia, 42.9%, all of them led to treatment discontinuation. These differences are likely to result from a stricter protocol for recording AEs in clinical trials [10] and the greater exhaustiveness of AEs in retrospective chart reviews compared to disease registries [28]. In PALOMA-2 trial, leukopenia, fatigue, nausea, arthralgia, and alopecia were also common. We have not reported such AEs as our data source only focuses on AEs leading to treatment discontinuation. Therefore, AEs identified were more severe and included mostly hematological effects.

This study has some limitations, namely the limited sample size in comparison to PALOMA-2 [10]. However, our sample size is in line with previous publications [19], and considerably higher than other real-world studies [16]. There were 8.4% of patients lost to follow-up, even though acceptable in oncology [29] and rarely quantified in effectiveness studies [18, 19]. Nonetheless, this population-based study included all patients registered at RON that were treated with the drug of interest in Portugal during the study period, an external validity advantage when compared to previous single-center retrospective cohort studies [16]. Early access use, might lead to channeling bias, resulting in underestimation of outcomes. The retrospective nature of the study could affect the estimation of outcomes, particularly PFS. In PALOMA-2, progression was evaluated every 12 weeks (± 7 days), and in a real-world context such evaluations are usually less frequent, which may lead to an overestimation of PFS. Finally, although it would be relevant to assess quality of life, as described elsewhere [12], this was not pursued as it is not routinely collected. Despite these limitations, considering the nature of the study, resorting to a population-based registry, the ability to have an extended follow-up period and the limited number of patients lost to follow-up, warrants some strength in findings.

## Conclusion

Palbociclib in association with an AI had a favorable effectiveness in terms of PFS that varied according to patients’ prognostic characteristics (reaching 28.8 months when used in patients trying to emulate characteristics to those used in PALOMA-2). The proportion of patients discontinuing treatment due to AEs was similar to those found in PALOMA-2.

## Supplementary Information

**Additional file 1**: **Table S1**. Characterization of lines of therapy received prior to exposure to palbociclib and AI. **Table S2**. Sensitivity analysis for progression-free survival. **Table S3**. Subgroup analysis for progression-free survival.

## Data Availability

The original contributions presented in the study are included in the article/Additional file. Further inquiries can be directed to the corresponding author.

## Abbreviations

ABC: Advanced breast cancer
AEs: Adverse events
AI: Aromatase inhibitor
CDK4/6: Cyclin-dependent kinase 4/6
CI: Confidence interval
ECOG PS: Eastern cooperative oncology group performance status
EMA: European medicines agency
ER: Endocrine receptor
HER2: Human epidermal growth factor receptor 2
HTA: Health technology assessment
INFARMED, I.P.: National authority of medicines and health products
IQR: Interquartile range
Palbo + AI: Palbociclib in association with an aromatase inhibitor
SD: Standard deviation
LHRH: Luteinizing hormone releasing hormone
NA: Not available
OS: Overall survival
PFS: Progression-free survival
RANK/RANKL: Receptor activator of NF-κB ligand
RON: Registo Oncológico Nacional (National oncology register)
STROBE: STrengthening the Reporting of OBservational studies in Epidemiology
TPF: Time to palbociclib failure
TTNT: Time to next treatment
