# Supplementary material for: Effectiveness of palbociclib with aromatase inhibitors for the treatment of advanced breast cancer in an exposure retrospective cohort study: implications for clinical practice
Source: Breast Cancer Res. 2023 Jun 29;25:78. doi: 10.1186/s13058-023-01678-5 (PMC10308630; doi:10.1186/s13058-023-01678-5)
Supplement: Supplementary file 1 — Additional file 1: Table S1. Characterization of lines of therapy received prior to exposure to palbociclib and AI. Table S2. Sensitivity analysis for progression-free survival. Table S3. Subgroup analysis for progression-free survival. [file 13058_2023_1678_MOESM1_ESM.docx]

**Supplementary Table S1-** Characterization of lines of therapy received prior to exposure to palbociclib and AI

| Previous treatments received | One line | Two lines | Three of more lines |
| --- | --- | --- | --- |
| Adjuvant endocrine therapy for locoregional disease at diagnosis (%)  Total 70 (53.4 %) | 55 (78.6%) | 12 (17.1%) | 3 (4.3%) |
| tamoxifen | 42 | 11 | 5 |
| letrozole | 4 | 7 | 3 |
| anastrozole | 5 | 4 | 1 |
| exemestane | - | 2 | - |
| Unspecified endocrine therapy | 4 | - | - |
| Endocrine therapy for advanced disease (%)  Total 23 (17.6%) | 14 (60.9%) | 7 (30.4%) | 2 (8.7%) |
| tamoxifen | 7 | 1 | 1 |
| letrozole | 2 | 6 | 1 |
| anastrozole | 2 | - | - |
| exemestane | 2 | 1 | - |
| fulvestrant | 1 | 6 | 4 |
| Chemotherapy for advanced disease (%)  Total 25 (19.1%)* | 12 (48.0%) | 9 (36.0%) | 4 (16.0%) |
| doxorubicin and cyclophosphamide | 2 | 3 | 2 |
| paclitaxel | 3 | 3 | 1 |
| capecitabine | 2 | 2 | 1 |
| epirubicin and cyclophosphamide | - | 2 | - |
| fluorouracil, epirubicin and cyclophosphamide | 1 | 2 | - |
| cyclophosphamide and methotrexate | 1 | - | - |
| docetaxel and epirubicin | - | - | 3 |
| capecitabine and paclitaxel | - | 1 | - |
| docetaxel | 1 | 3 | 1 |
| vinorelbine | - | - | 1 |
| capecitabine and vinorelbine | - | - | 1 |
| epirubicin | - | 1 | - |
| eribulin | - | 1 | - |
| doxorubicin | - | - | 1 |

*There were two patients included in a clinical trial; however, considering the blinding process, we have not considered them in the description of chemotherapy.

**Supplementary Table S2– Sensitivity analysis for progression-free survival**

|  | **Number of events / n (%)** | **Median (95%CI)** | **1-year rate (95%CI)** | **2-year rate (95%CI)** |
| --- | --- | --- | --- | --- |
| **PFS sensitivity analysis** | | | | |
| Excluding patients with an initial palbociclib dose of 100mg 75mg | 80/118 (67.8) | 19.8 months (14.4 – 28.9) | 69.5%  (60.3 – 77.0) | 43.2% (34.2 – 51.9) |
| Excluding patients who switched to another AI in the course of treatment | 89/130 (68.5) | 19.4 months (14.2 – 23.2) | 67.7%  (58.9 – 75.0) | 41.5% (33.0 – 49.8) |
| Excluding patients with clinically evaluated progression (i.e., only patients with radiological evaluation) | 74/115 (64.4) | 21.2 months (15.4 – 30.0) | 72.2% (63.0 – 79.4) | 46.1%  (36.8 – 54.9) |
| Excluding patients with 1) previous exposure to at least one systemic treatment line to advanced disease, or 2) disease recurrence while receiving neoadjuvant or adjuvant therapy with AI or within 12 months after completing this therapy | 58/91 (63.7) | 23.0 months  (16.8 – 32.6) | 73.6%  (63.3 – 81.5) | 48.4%  (37.8 – 58.1) |
| Excluding patients with pre-menopausal status | 62/88 (70.5) | 19.7 months (12.6 – 25.9) | 63.6% (52.7 – 72.7) | 40.9% (30.6 – 50.9) |
| All 5 criteria simultaneously (*35.1% of total number of patients*) | 27/46 (58.70) | 28.8 months (19.4 – 36.0) | 82.6% (68.2 – 90.9) | 54.4% (39.0 – 67.4) |

Legend: CI, confidence interval; TPF, time-to-palbociclib failure; PFS, Progression-free survival

**Supplementary Table S3 - subgroup analysis for progression-free survival**

| Subgroup | Progression events, n (%) | Median PFS (95% CI), months | 1-year PFS rate, % | 2-years PFS rate, % |
| --- | --- | --- | --- | --- |
| Stage IV at diagnosis (n=51) | 29 (56.9) | 20.2 (12.9 – NR) | 72.6 (58.1 – 82.7) | 47.1 (33.0 – 59.9) |
| Stages I-III at diagnosis (n=80) | 61 (76.3) | 18.0 (13.0 – 23.2) | 65.0 (53.5 – 74.3) | 38.8 (28.2 – 49.2) |
| Visceral metastases (with or without bone involvement) (n=72)* | 52 (72.2) | 15.5 (11.2 – 23.0) | 61.1 (48.9 – 71.3) | 38.9 (27.7 – 49.9) |
| Only bone metastases (n=38)* | 25 (65.8) | 20.4 (14.7 – 36.3) | 79.0 (62.3 – 88.9) | 44.7 (28.7– 59.6) |

Legend: NR, not reached; *non-visceral metastases are not presented as the subgroup dimension was ≤ 30
